# Circulating tumor cells detected in follow-up predict survival outcomes in tri-modality management of advanced non-metastatic esophageal cancer: a secondary analysis of the QUINTETT randomized trial

**DOI:** 10.1186/s12885-022-09846-0

**Published:** 2022-07-08

**Authors:** Edward Yu, Alison L. Allan, Michael Sanatani, Debra Lewis, Andrew Warner, A. Rashid Dar, Brian P. Yaremko, Lori E. Lowes, David A. Palma, Jacques Raphael, Mark D. Vincent, George B. Rodrigues, Dalilah Fortin, Richard I. Inculet, Eric Frechette, Joel Bierer, Jeffery Law, Jawaid Younus, Richard A. Malthaner

**Affiliations:** 1 Department of Oncology, Divisions of Radiation Oncology, Western University, 1151 Richmond Street, London, Ontario N6A3K7 Canada; 2 Experimental Oncology, London, Ontario Canada; 3 Medical Oncology, London, Ontario Canada; 4 Thoracic Surgery and Surgical Oncology, London, Ontario Canada; 5 Pathology & laboratory medicine, London Health Science Centre, London, Ontario Canada; 6 Department of Thoracic Surgery and Surgical Oncology, Sherbrooke University, Sherbrooke, Quebec Canada; 7 Department of Medicine, Western University, London, Ontario Canada

**Keywords:** Circulating tumor cells, Prognostic, Non-metastatic, Esophageal cancer

## Abstract

**Background:**

Our aim was to establish if presence of circulating tumor cells (CTCs) predicted worse outcome in patients with non-metastatic esophageal cancer undergoing tri-modality therapy.

**Methods:**

We prospectively collected CTC data from patients with operable non-metastatic esophageal cancer from April 2009 to November 2016 enrolled in our QUINTETT esophageal cancer randomized trial (NCT00907543). Patients were randomized to receive either neoadjuvant cisplatin and 5-fluorouracil (5-FU) plus radiotherapy followed by surgical resection (Neoadjuvant) or adjuvant cisplatin, 5-FU, and epirubicin chemotherapy with concurrent extended volume radiotherapy following surgical resection (Adjuvant). CTCs were identified with the CellSearch® system before the initiation of any treatment (surgery or chemoradiotherapy) as well as at 6-, 12-, and 24-months post-treatment. The threshold for CTC positivity was one and the findings were correlated with patient prognosis.

**Results:**

CTC data were available for 74 of 96 patients and identified in 27 patients (36.5%) at a median follow-up of 13.1months (interquartile range:6.8-24.1 months). Detection of CTCs at any follow-up visit was significantly predictive of worse disease-free survival (DFS;hazard ratio [HR]: 2.44; 95% confidence interval [CI]: 1.41-4.24; *p*=0.002), regional control (HR: 6.18; 95% CI: 1.18-32.35; *p*=0.031), distant control (HR: 2.93; 95% CI: 1.52-5.65;*p*=0.001) and overall survival (OS;HR: 2.02; 95% CI: 1.16-3.51; *p*=0.013). After adjusting for receiving neoadjuvant vs. adjuvant chemoradiotherapy, the presence of CTCs at any follow-up visit remained significantly predictive of worse OS ([HR]:2.02;95% [Cl]:1.16-3.51; *p*=0.013) and DFS (HR: 2.49;95% Cl: 1.43-4.33; *p*=0.001). Similarly, any observed increase in CTCs was significantly predictive of worse OS (HR: 3.14; 95% CI: 1.56-6.34; *p*=0.001) and DFS (HR: 3.34; 95% CI: 1.67-6.69; *p*<0.001).

**Conclusion:**

The presence of CTCs in patients during follow-up after tri-modality therapy was associated with significantly poorer DFS and OS regardless of timing of chemoradiotherapy.

## Introduction

The identification of patients at higher risk of recurrence after treatment remains one of the most elusive challenges in clinical oncology. Circulating tumor cells (CTCs) may represent an indicator of more extensive disease in patients without overt metastases. CTCs in the peripheral blood of patients were first documented almost 150 years ago and represent the hematogenous spreading of cells from a primary tumor [1]. The shedding of these cells makes up a critical step in the metastatic cascade, ultimately leading to the establishment of distant metastases [2]. Recent advances in technology have allowed for the sensitive and specific identification of CTCs in peripheral blood samples through the expression of epithelial and cancer specific markers. The CellSearch® system (Menarini Silicon Biosystems, Philadelphia, PA) is currently the only US Food and Drug Administration (FDA) and Health Canada approved assay for CTC analysis and has also been approved for routine clinical use in metastatic breast, prostate, and colon cancer [3]. Over the past number of years, CTCs have been found to provide valuable information in prognosis and treatment outcomes in these and other cancer types [4–7]_._

Historically, most data on the incidence and role of CTCs have been derived from studies conducted in metastatic cancer. The correlation between number of CTCs and increased patient mortality rates is well documented in several metastatic cancers including breast, colorectal and prostate [3–6]. Similarly, the level of CTCs in esophagogastric cancer appears to also predict tumor prognosis, however, there have been significant limitations in data including relatively fewer studies, a variety of methods used for CTC detection, and many of the studies were performed in East Asian populations [8–20]. However, to our knowledge, there has been limited formal study assessing CTCs as a prognostic factor in patients with non-metastatic esophageal cancer in the tri-modality setting [21]. Specially, prior to the present study, it was unknown whether CTCs could be detected in this patient population and whether these cells carry prognostic significance. Finally, while there have been studies investigating levels of CTCs in cancer treatment, there has never been a head-to-head randomized clinical trial assessing whether the presence of CTCs predicted worse outcome in patients with non-metastatic esophageal cancer and if any differences exist between adjuvant or neoadjuvant treatment approaches.

Using the CellSearch® immunomagnetic CTC detection platform, we present a prospective study designed to investigate whether CTCs can predict outcomes in chemotherapy naïve patients with non-metastatic esophageal cancer. Our goals for the study were to investigate whether CTCs (1) can be detected in non-metastatic esophageal cancer patients receiving tri-modality therapy, and (2) have value in predicting prognosis. We hypothesized that CTCs within the blood would be associated with worse disease-free survival (DFS) and overall survival (OS), ultimately providing information that could be useful in disease staging and in identifying patients who might benefit from specialized treatment strategies.

## Methods

### Study design

**Fig. 1:**
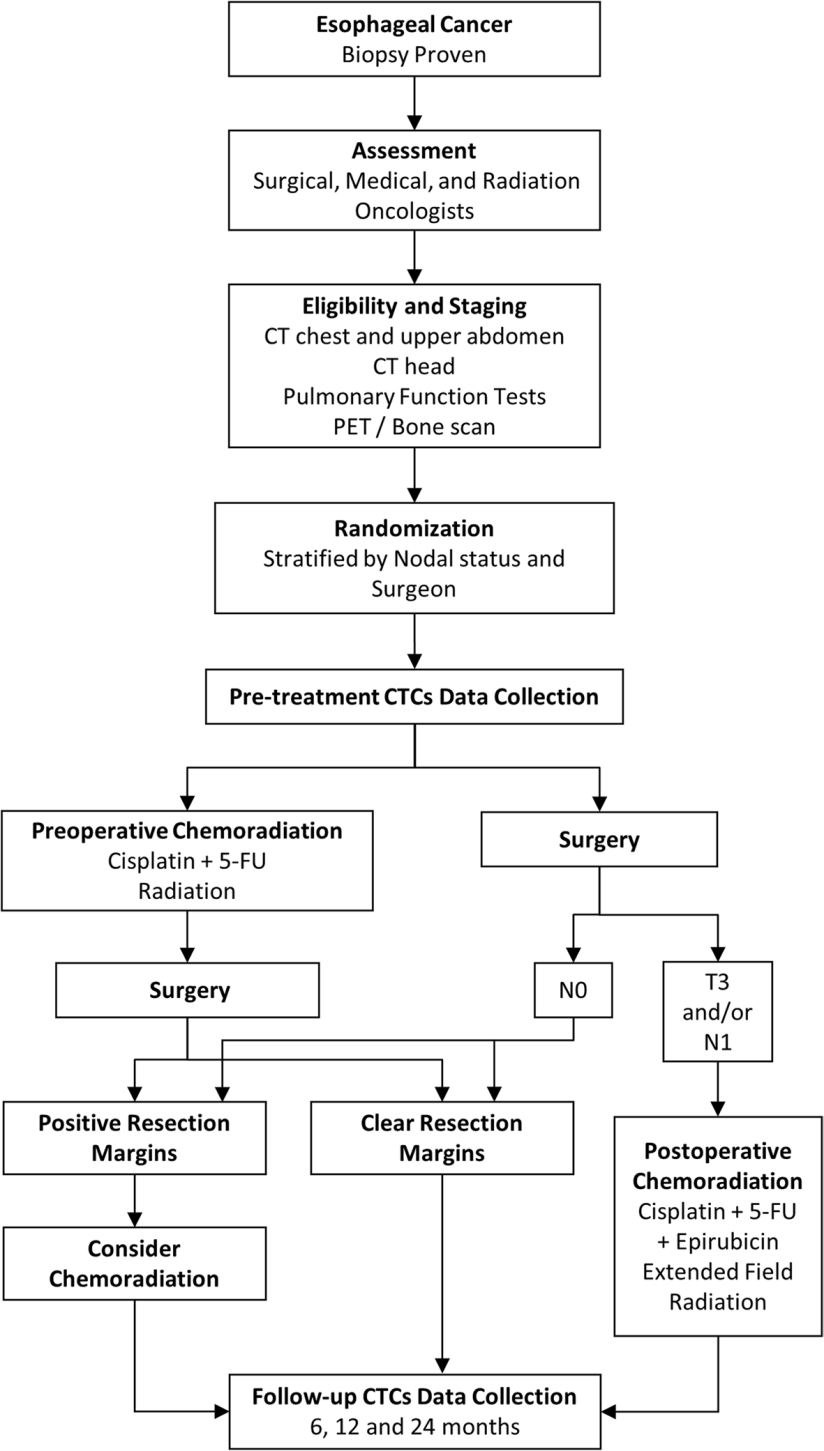
Study design. Abbreviations: CT – computed tomography; PET – positron emission tomography; CTCs – circulating tumor cells; 5-FU – 5-fluorouracil

The Quality of Life In Neoadjuvant vs. Adjuvant Therapy of Esophageal Cancer Treatment Trial (QUINTETT) was a prospective, randomized superiority trial enrolling patients between April 2009 and November 2016 (NCT00907543), summarized in Fig. 1. The study was completed and approved by our Institutional Ethics Review Board (#15817) and all included patients provided written informed consent. The trial was supported by local internal institutional grants with no external or industry sponsorship.

### Patients

Patients with stage I to III, T1-3, N0-1resectable esophageal cancer were sequentially screened at our institution and referred to the study. All patients were preoperatively assessed with a history, physical examination, haematologic and biochemical testing, pulmonary function testing, computed tomography (CT) scan of the chest and upper abdomen, bone scan, CT scan of the head and esophagogastroduodenoscopy (EGD) at minimum. Patients were required to be chemotherapy naïve with histologically documented squamous cell carcinoma or adenocarcinoma of the thoracic esophagus (> 20 cm from the incisors) or gastroesophageal junction (< 3 cm into the stomach) undergoing surgery for their primary tumor. Patients with distant metastases (M1), cancers of the cervical esophagus (≤ 20 cm), or any other malignancy within 5 years of diagnosis of the present cancer were ineligible. Informed consent was obtained prior to collection of blood samples. A positron emission tomography (PET) scan replaced the bone scan if possible.

### Randomization

Per QUINTETT trial [22] patients were stratified by clinical nodal status (N0 vs. N1) and by surgeon and then randomized 1:1 to either Neoadjuvant treatment arm (control) or Adjuvant treatment arm (intervention). Randomization occurred centrally through the Thoracic Clinical Trials office to ensure concealment of the process.

### Surgery, chemotherapy and radiation therapy

As described in detail in the QUINTETT trial [22], in brief, transhiatal esophagectomy was performed for lower third lesions. Our current standard is a minimally invasive approach (video-assisted thoracoscopic surgery [VATS] and/or laparoscopic) as per tumor location and/or surgeon preference.

For chemotherapy, the Neoadjuvant arm, treatment began within 4 weeks of randomization consisting of two cycles of induction cisplatin 25 mg/m^2^on days 1, 2, 3, and 4 concurrent with 5-fluorouracil (5-FU) 1000 mg/m^2^ per day for 96 hours continuous venous infusion for two cycles every 28 days, administered to coincide as closely as possible with the start of concurrent radiation therapy. In the Adjuvant arm, treatment began between 8- and 12- weeks following surgery. Chemotherapy was administered alone for 2 cycles; one cycle consisted of epirubicin 50 mg/m^2^ and cisplatin 60 mg/m^2^on day 1, and 5-FU by continuous venous infusion at 200 mg/m^2^ for 21 days. Chemotherapy (concurrent with radiation) was given immediately afterwards for two additional cycles; one cycle consisted of cisplatin 60 mg/m^2^on day 1, and 5-FU by continuous venous infusion at 200 mg/m^2^ for 21 days. Concurrent radiation was started on the same day as the third cycle if possible, but in all cases within 3 days before or after that date.

Radiation therapy was administered using three-dimensional planning technology including intensity modulated radiation therapy (IMRT). The total dose for both arms was 50.4Gy delivered in 28 fractions (1.8 Gy dose per fraction), 5 days a week. In the Neoadjuvant arm, radiation was adopted with minor modifications from RTOG 0113 [23], whereas in the Adjuvant arm, radiation was delivered as previously described by Yu *et al.* [24].

### Blood samples and CTC detection

A total of 74 of 96patients enrolled in QUINTETT participated in this correlative study and were prospectively evaluated for the presence of CTCs. After randomization, we collected peripheral blood (7.5 mL) at the time of patient consent prior to the initiation of any treatment (surgery or chemoradiotherapy) as well as at 6-, 12-, and 24-months post-treatment. All blood samples were collected into 10 mLCellSave® Preservative Tubes (Menarini Silicon Biosystems, Philadelphia, PA). The samples were maintained at room temperature and processed within 96 hours after collection according to the guidelines of the manufacturer.

For isolation and enumeration of CTCs, the CellSearch® Circulating Tumor Cell Test (Menarini Silicon Biosystems, Philadelphia, PA) was used. This assay consists of a semi-automated system where cells expressing epithelial-cell adhesion molecule (EpCAM) on their cell membranes are enriched by immunomagnetic separation using ferro-fluids coated with EpCAM antibodies. The isolated cells were fluorescently labeled with the nucleic acid dye 4,2-diamidino-2-phenylindole dihydrochloride (DAPI) and a monoclonal antibody against cytokeratin 8, 18, and 19. A monoclonal antibody against cluster of differentiation 45 antigen (CD45, a common leukocyte antigen) were used to distinguish leukocytes from epithelial cells. Further identification and enumeration of CTCs was performed by the CellTracks® Analyzer II, a semi-automated fluorescence microscopy system. Finally, a gallery of cellular images was reviewed and interpreted independently by two trained readers from whom all patient data was masked. CTCs were defined as nucleated cells (DAPI-positive) staining positive for cytokeratin and negative for CD45 and showing cellular size (> 4 μm) and morphology in keeping with viable, intact tumor cells. The results were expressed as the number of CTCs per 7.5 mL of peripheral blood. The threshold for CTC positivity was one [25].

### Endpoints

For the present study based on a secondary correlative analysis of CTCs, only OS and DFS secondary endpoints were examined and reported. OS and DFS, both calculated from date of randomization to date of recurrence (DFS only), date of death due to any cause, or date of last follow-up, whichever occurs first.

### Statistical analysis

Descriptive statistics were generated for patient characteristics for all patients and stratified by treatment arm, compared using the chi-square test, Fisher’s exact test, two-sample t-test or Wilcoxon rank sum test as appropriate. Univariable Cox proportional hazards regression was performed for OS and DFS for all eligible variables for all patients. All models were stratified by clinical nodal status (N0 vs. N1) and adjusted for treatment arm. Kaplan-Meier estimates were generated for OS and DFS for all patients and stratified by treatment arm, CTCs at any follow-up visits, and CTCs at 6-month follow-up, compared using the stratified log-rank test stratified by clinical nodal status (N0 vs. N1). All statistical analysis was intention-to-treat and performed using SAS version 9.4 software (SAS Institute, Cary, NC, USA) using two-sided statistical testing at the 0.05 significance level.

## Results

**Fig. 2:**
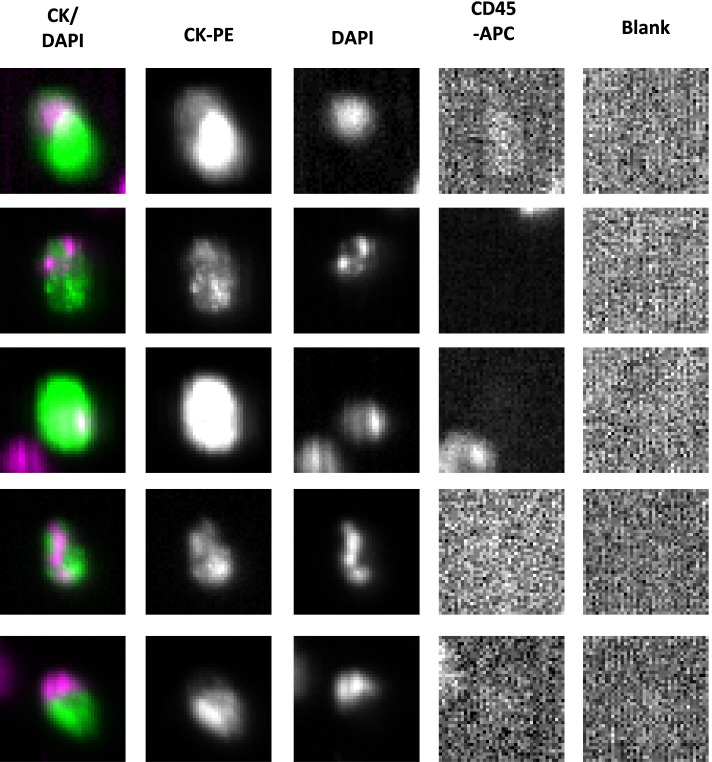
Representative images of circulation tumor cells (CTCs) captured using the CellSearch® System

Ninety-six patients were randomized in QUINTETT, 47 patients to Neoadjuvant arm and 49 patients to Adjuvant arm. Patient characteristics are summarized and results from QUINTETT based on all 96 enrolled patients have been reported previously [22]. For the secondary correlative analysis of CTCs, 74 (77.1%) patients had CTC data available and were included in this analysis. Twenty-seven (36.5%) patients had detectable CTCs at any follow-up visit, 13(37.1%) in Neoadjuvant arm and 14(35.9%) in Adjuvant arm. CTC counts were also examined 6-, 12-, and 24-months post-treatment. At baseline, mean ± SD CTC counts were 6.5 ± 45.1 (range: 0-380; Table 1). Median follow-up for CTC data collection was 13.1months (interquartile range:6.8-24.1months) (Fig. 2).

**Table 1:** Summary of CTC measurements

Characteristic	N	All Patients (*n*=96)	Neoadjuvant CRT Arm (*n*=47)	Adjuvant CRT Arm (*n*=49)	*p*-value
**Any CTCs**^a^– n(%)					
Any	74	27 (36.5)	13 (37.1)	14 (35.9)	0.912
Baseline	72	16 (22.2)	9 (26.5)	7 (18.4)	0.412
6 months	53	6 (11.3)	5 (20.0)	1 (3.6)	0.089
1 year	48	11 (22.9)	5 (23.8)	6 (22.2)	> 0.99
2 years	29	6 (20.7)	4 (26.7)	2 (14.3)	0.651
**CTC count** – mean ± SD					
Baseline	72	6.5 ± 45.1	2.1 ± 9.6	10.5 ± 61.6	0.504
6 months	53	0.7 ± 2.8	1.0 ± 2.9	0.5 ± 2.8	0.078
1 year	48	2.4 ± 14.0	4.9 ± 21.1	0.4 ± 1.0	0.888
2 years	29	5.1 ± 17.0	9.7 ± 23.0	0.2 ± 0.6	0.309
**CTC count change** – mean ± SD					
6 months vs. baseline	51	0.2 ± 3.1	0.5 ± 2.7	0.0 ± 3.4	0.488
1 year vs. baseline	46	1.9 ± 13.5	4.5 ± 20.4	-0.1 ± 1.9	0.743
2 years vs. baseline	28	4.6 ± 17.4	9.4 ± 22.9	-0.8 ± 2.5	0.162
**CTC follow-up (months)** – median (IQR)	74	13.1 (6.8, 24.1)	17.9 (6.8, 23.9)	12.7 (6.2, 24.3)	0.837

*CTC* Circulating tumor cell, *CRT* Chemoradiotherapy, *IQR* Interquartile range

^**a**^Categories not mutually exclusive

**Fig. 3:**
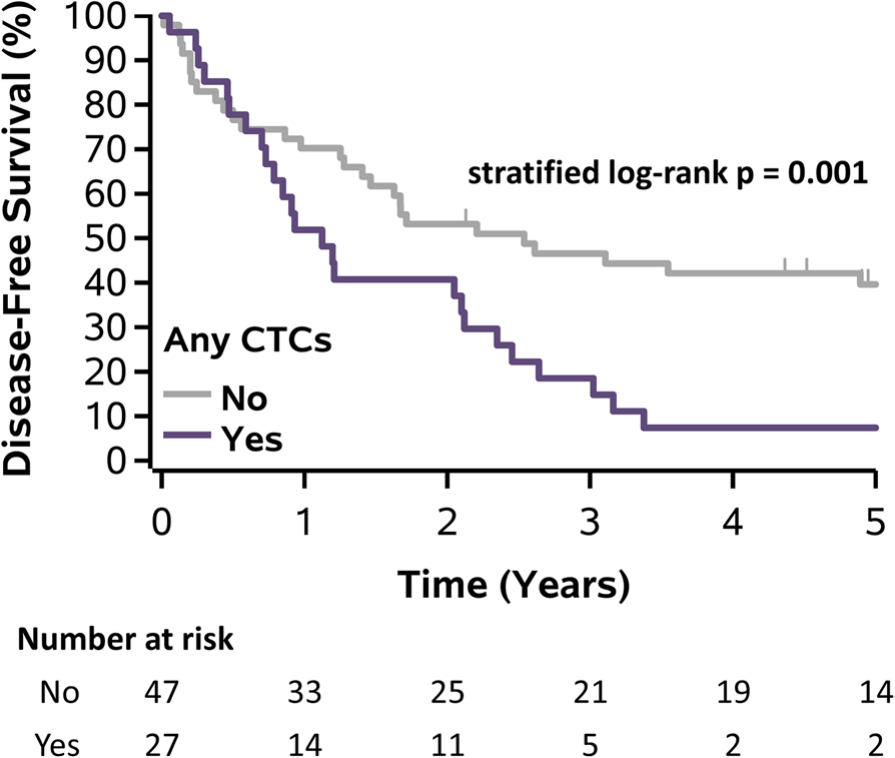
Kaplan-Meier plot of overall survival for all patients stratified by CTCs at any follow-up visits. Abbreviations: CTCs – circulating tumor cells.

**Fig. 4:**
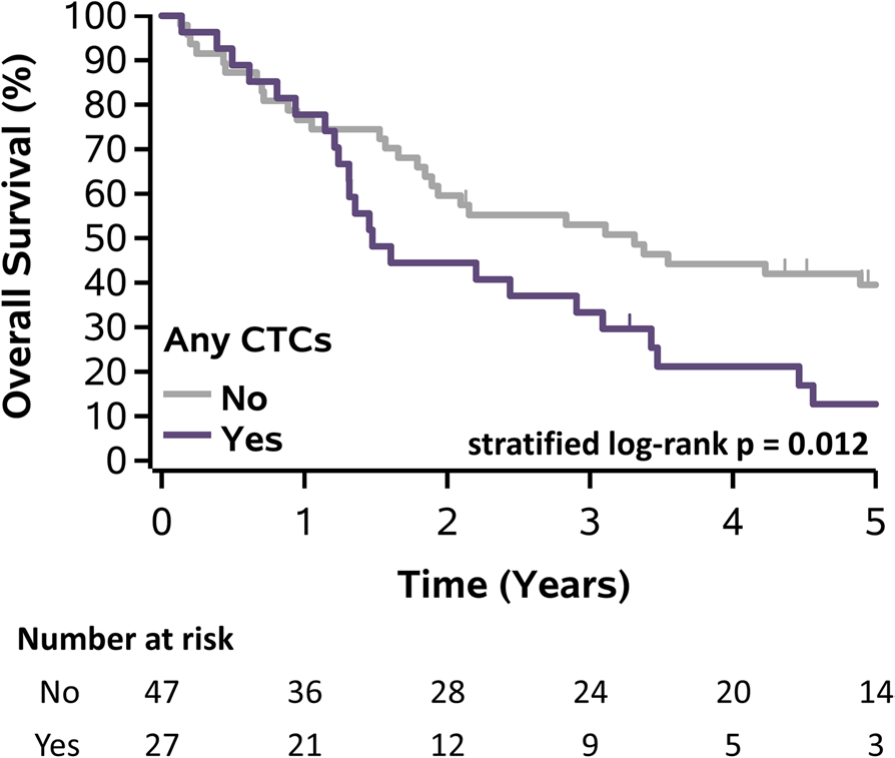
Kaplan-Meier plot of disease-free survival for all patients stratified by CTCs at any follow-up visits. Abbreviations: CTCs – circulating tumor cells

Patients with CTCs detected at baseline prior therapy vs. no CTCs had a significant poor prognostic outcome with DFS (median DFS: 0.92 years vs 1.88 years; *p*=0.023), but not OS ( median OS:1.46 years vs 2.44 years, *p*=0.064). Patients with CTCs detected at any follow-up visits vs. no CTCs had worse OS (median OS: 1.48 years vs. 3.31 years; *p*=0.012), and DFS (median DFS: 1.12 years vs. 2.54 years; *p*=0.001) (Figs. 3 and 4, respectively). This translated to a hazard ratio (HR) of 2.02(95% confidence interval [CI]: 1.16-3.51; *p*=0.013) for OS and HR of 2.44 (95% CI: 1.41-4.24, *p*=0.002) for DFS. For Neoadjuvant arm, patients with CTCs detected at any follow-up visits had significantly worse DFS (median DFS: 0.93 years vs. 2.54 years; *p*=0.030) but not OS (median OS: 1.48 years vs. 3.11 years; *p*=0.142). For Adjuvant arm, patients with CTCs had worse OS (median OS: 1.46 years vs. 3.31 years; *p*=0.078) and DFS (median DFS: 1.16 years vs. 2.21 years; *p*=0.056), but neither were significant.

Similarly, patients with CTCs detected at 6-month follow-up vs. no CTCs had worse OS (median OS: 1.30 years vs. 3.55 years; *p*=0.213) and DFS (median DFS: 0.71 years vs. 3.02 years; *p* < 0.001). For Neoadjuvant arm, patients with CTCs detected at 6-month follow-up had significantly worse DFS (median DFS: 0.73 years vs. 2.54 years; *p*=0.003) but not OS (median OS: 1.45 years vs. 3.43 years; *p*=0.630). For Adjuvant arm, no comparisons were made due to only 1 patient having CTCs at 6-month follow-up.

There were 13 (17.6%) patients observed to have a decrease in CTC number in follow-up post-treatment;7 (20.0%) in the Neoadjuvant arm and 6 (15.4%) in the Adjuvant arm. In contrast, there were 14 (18.9%) patients observed to have an increase or no change in CTC number in follow-up post-treatment;6 (17.1%) in the Neoadjuvant arm and 8(20.5%) in the Adjuvant arm.

After adjusting for treatment arm (receiving neoadjuvant vs. adjuvant chemoradiation) and stratifying by clinical nodal status (N0 vs. N1), the presence of CTCs at any follow-up visit remained significantly predictive of worse OS ([HR]: 2.02;95%[CI]: 1.16-3.51; *p*=0.013) and DFS (HR:2.49; 95%CI: 1.43-4.33; *p*=0.001). Any observed increase in CTC number was significantly predictive of worse OS (HR: 3.14; 95% CI: 1.56-6.34; *p*=0.001) and DFS (HR: 3.34; 95% CI: 1.67-6.69; p<0.001).

## Discussion

Our study reported that the presence of CTCs in patients after tri-modality therapy was associated with significantly poorer DFS and OS regardless of timing of chemoradiotherapy. Our study patients were screened with PET if possible, instead of a bone scan, to ensure no metastatic disease at the start of treatment. This is the first report on the significance of CTCs in non-metastatic esophageal cancer patients undergoing tri-modality therapy.

CTCs have been detected over the years through several means, including immunocytochemistry, reverse transcription polymerase chain reaction (RT-PCR) and flow cytometry [26–28]. Direct comparisons between studies are challenging due to heterogeneity in laboratory techniques used to detect the CTCs. For example, RT-PCR is a more sensitive test than immunocytochemistry, but the specificity may be hindered by high numbers of false positive results due to contamination or target genes expressed in non-malignant cells and the amplification-based nature of PCR [29–32]. However, a commercially available assay has been developed (CellSearch®, Circulating Tumor Cell Test, Menarini Silicon Biosystems) and approved by the US FDA and Health Canada for the detection of CTCs. This method has been validated, found to be reproducible and is the method used in this study [33–37].

The detection of CTCs through CellSearch® in non-metastatic disease may allow for the identification of high-risk patients who may require additional therapy, whether it be adjuvant therapy where not previously indicated or more aggressive chemoradiation. Most studies that have investigated CTCs in non-metastatic cancer have been in the context of breast, colorectal and prostate cancer. Fewer data are available on the detection and prevalence of CTCs in non-metastatic esophageal cancer. For metastatic esophageal cancer, Sclafani *et al.* found that ≥2 CTCs were detected in 44% (8/18) of patients investigated [38]. In non-metastatic gastrointestinal cancer patients, Hiraiwa*et al.* measured CTCs in 10 (29%) esophageal cancer patients on subgroup analysis and found no individuals to have ≥2 CTCs [20]. This is in contrast to our current study where 36.5% of patients (27/74) with non-metastatic esophageal cancer were found to be CTC positive. Such a difference can be attributed to the small sample size of the Hiraiwa study, but possibly due to potential variations in epidemiology and histology of esophageal cancers between Eastern and Western countries [38].

Most studies to date have correlated CTC counts with prognosis in metastatic disease at a point where treatment intent is palliative, or studies with non-metastatic disease treated with non tri-modality approach [21]. Whether CTCs hold prognostic significance in non-metastatic patients at a time when treatment can still be altered to maximize curative potential remains unclear. Sclafani *et al.* showed that having ≥2 CTCs per 7.5 mL blood predicted progression-free survival and OS in patients with metastatic esophagogastric cancers [39]. For non-metastatic a single prospective randomized clinical trial has just been completed and a few are currently ongoing [40–42]. Lucci *et al.* reported that ≥1 CTC before the start of therapy was an independent predictor of both progression-free survival and OS [4]. Our trial is currently the only prospective randomized clinical trial for non-metastatic esophageal cancer with tri-modality therapy. Our findings show that the presence of ≥1 CTC per 7.5 mL blood as a predictor of DFS and OS in chemotherapy naïve patients.

Recently Reech *et al.* [43] reported CTCs can be a biomarker for preoperative staging of esophageal cancer. CTCs in multivariable analyses were shown to be an independent prognostic indicator of tumor recurrence and OS. In addition, CTCs have been documented to play a role as a predictor of chemotherapy response. Sclafani *et al.* observed a decrease in the number of detected CTCs during chemotherapy in all patients with baseline CTCs ≥2 in metastatic esophagogastric cancers treated with combined epirubicin/oxaliplatin/capecitabine chemotherapy ± panitumumab [37]. In our study, patients were randomized to receive either neoadjuvant or adjuvant therapy in order to investigate any potential differences in CTC reduction.

For CTCs detected at any follow-up visit, we observed significantly worse OS (*p*=0.012) and DFS (*p*=0.001), however this was only significant for DFS for Neoadjuvant arm (*p*=0.030). For CTCs detected at 6-month follow-up, worse OS and DFS were observed but only significant for DFS (p < 0.001), most likely attributed to only 6 patients having CTCs at 6-month follow-up, as discussed previously, and similarly only significant for DFS for Neoadjuvant arm (*p*=0.003). For Adjuvant arm, unfortunately no comparisons could be made due to sample size limitations given only 1 patient had CTCs at 6-month follow-up. In addition to the smaller sample size and shorter follow-up of both treatment arms, there is a possibility that the potential presence of resistant clones of CTCs may have played a role. Studies have suggested that CTCs can exhibit putative cancer stem cell phenotypes associated with the upregulation of multidrug-resistance proteins [44–46].CTCs can also evolve to no longer express epithelial markers [47], which are necessary for detection by CellSearch®.CTCs can undergo an epithelial mesenchymal transition (EMT) process and exhibit the characteristics of stem cells, escaping hematological detection by down-regulating their epithelial markers and up-regulating their mesenchymal markers, leading to false negative results [48]. Chen *et al.* have reported mesenchymal CTCs by measuring vimentin and Twist, correlating their presence in late stage squamous cell esophageal cancer [49].

We acknowledge several limitations in our study, including secondary analysis of a randomized trial, small sample size, rarity of CTCs in non-metastatic patient population, and short follow up. We also acknowledge that the CTCs detection system relied on CTCs epithelial markers procession, cell homogeneity, and potential difference of detection between adenocarcinoma and squamous cell carcinoma due to difference in expression of EpCAM. In our view these limitations do not detract from the reliability of our result that the presence of CTCs detected following curative treatment was associated with poor patient outcome.

Currently, there is no recommendation for the use of CTCs in the decision-making process for patients with esophageal cancer. However, the growing body of published work [50], including our study, suggests CTCs may provide important prognostic information to assist identification of patients at risk of disease recurrence to benefit further therapeutic treatment [51]_._ Our findings showed that CTCs can be potential prognostic indicator in follow-up post tri-modality treatment. Our data also support baseline CTCs prior therapy can be potential prognostic indicator. The use of adjuvant immunotherapy has been shown to be beneficial [52]. Our plan will be to further investigate the potential benefit from modification of systemic and immunotherapy treatment with the assistance of CTCs as prognostic indicator in a tri-modality approach, either in adjuvant or neoadjuvant setting [53]. The University Health Network in Toronto, Ontario, Canada, is conducting a clinical trial on the utility of CTCs and plasma microRNA in esophageal adenocarcinoma (NCT02812680) estimated to be completed by 2023.The result of these trials may provide further insight on the role of CTCs in esophageal cancer management.

## Conclusion

To our knowledge, this is the first study to report that CTC detection by CellSearch® system is prognostic inpatients with non-metastatic esophageal cancer treated with tri-modality therapy. Patients with positive CTCs during follow-up post tri-modality therapy were associated with significantly poorer DFS and OS regardless of timing of chemoradiotherapy.

## Data Availability

The datasets used and analysed during the current study available through the corresponding author with the study team and LHRI Ethics Board permissions only on reasonable request.
